# Near-Infrared Reflectance Imaging in Retinal Diseases Affecting Young Patients

**DOI:** 10.1155/2021/5581851

**Published:** 2021-07-31

**Authors:** Solmaz Abdolrahimzadeh, Chiara Ciancimino, Flaminia Grassi, Edoardo Sordi, Serena Fragiotta, Gianluca Scuderi

**Affiliations:** Ophthalmology Unit, “Sapienza” University of Rome, NESMOS Department, St. Andrea Hospital, Via di Grottarossa 1035/1039, Rome, Italy

## Abstract

Near-infrared reflectance (NIR) is a noninvasive, contactless, and rapid in vivo imaging technique for visualizing subretinal alterations in the photoreceptor layer, retinal pigment epithelium, and choroid. The present report describes the application of this imaging method in retinal and choroidal pathologies affecting young patients where scarce cooperation, poor fixation, and intense glare sensation can result in a challenging clinical examination. A literature search of the MEDLINE database was performed using the terms “near-infrared reflectance” and “spectral-domain optical coherence tomography.” Articles were selected if they described the diagnostic use of NIR in children or young adults. Of 700 publications, 42 manuscripts published between 2005 and 2020 were inherent to children or young adults and were considered in this narrative literature review. The first disease category is the phakomatoses where NIR is essential in visualizing choroidal alterations recognized as cardinal biomarkers in neurofibromatosis type 1, microvascular retinal alterations, and retinal astrocytic hamartomas. Another diagnostic application is the accurate visualization of crystals of various nature, including the glistening crystals that characterize Bietti crystalline dystrophy. Acute macular neuropathy and paracentral acute middle maculopathy represent a further disease category with young adulthood onset where NIR is not only diagnostic but also essential to monitor disease progression. A further interesting clinical application is to facilitate the detection of laser-induced maculopathy where funduscopic examination can be normal or subnormal. In conclusion, NIR imaging has a noninterchangeable role in diagnosing certain retinal diseases, especially in children and young adults where there is scarce collaboration and a lack of evident clinical findings. Moreover, this technique can reveal unique retinal and choroidal biomarkers highly specific to rare conditions.

## 1. Introduction

Near-infrared reflectance (NIR) imaging is a noninvasive, noncontact, and rapid in vivo examination currently extensively available for ophthalmologists. Most images are acquired simultaneously with cross-sectional spectral-domain optical coherence tomography (SDOCT) in routine clinical practice. Specifically, the technique enables visualizing subretinal alterations located in the retinal photoreceptor layer, the retinal pigmented epithelium (RPE), and choroid [1]. The basis is a long excitation wavelength (∼820 nm diode laser) that penetrates the optic media and enables visualizing the retina and choroid in detail [2]. Changes in reflection and absorption of light through retinal tissues enhance visualization of structures beneath the retinal pigment epithelium (RPE) and melanin [1, 3]. Images often correlate with blue-light fundus autofluorescence (FAF); however, NIR demonstrates superiority in revealing sub-RPE lesions. This is due to a more substantial absorbance of monochromatic light of a shorter wavelength (480 nm) by melanin and lipofuscin granules at the RPE level [3, 4]. Highly reflective structures at the subretinal and sub-RPE level are enhanced and better recognized. For instance, hyperreflective crystalline deposits representing cholesterol crystals appear as intensely reflective plaques, while calcifications or calcified drusen are appreciated as roundish lesions with a glistening appearance [5–7]. The glistening appearance is also characteristic of crystalline deposits in Bietti crystalline dystrophy [8]. Such similarities in reflectance between inherited retinal dystrophy and age-related changes can also be seen in retinitis punctata albescens and reticular pseudodrusen [9].

NIR has gained widespread use in ophthalmology in the last decade. The present report underlines the importance of this imaging method in retinal and choroidal pathologies affecting young patients where scarce cooperation, poor fixation, and intense glare sensation can make fundus examination challenging.

## 2. Methods

A literature search of the MEDLINE database was performed using the terms “near-infrared reflectance (NIR)” and “spectral-domain optical coherence tomography” for articles in English accessed through December 2020. The articles were selected if they described the diagnostic use of NIR in children or young adults. Of 700 publications, 42 manuscripts published between 2005 and 2020 were inherent to children or young adults and are described in this narrative literature review. Reference lists of the selected manuscripts were also analysed to retrieve other relevant studies.

## 3. Neuro-Oculocutaneous Syndromes/Phakomatosis

Neurofibromatosis type 1 (NF1), tuberous sclerosis complex (TSC), and Sturge–Weber syndrome (SWS) are neuro-oculocutaneous diseases often classified among the phakomatoses [10]. Multimodal imaging methods enable facilitated visualization of retinal and choroidal changes and have improved diagnostic and management strategies in these diseases.

### 3.1. Neurofibromatosis Type 1

**Figure 1:**
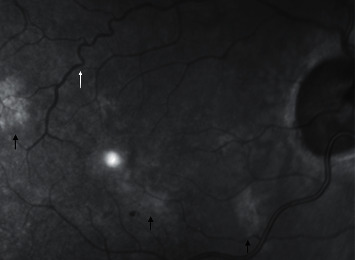
Near-infrared reflectance fundus image in neurofibromatosis type 1 (NF1). Hyperreflective choroidal alterations typical of NF1 are shown with black arrows, corkscrew retinal vascular alteration is shown with a white arrow.

**Figure 2:**
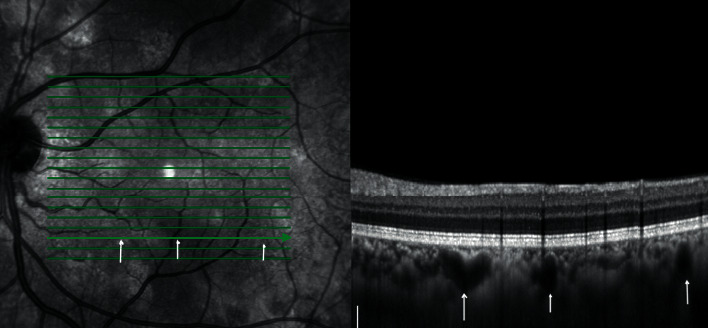
Near-infrared reflectance (NIR) and cross-sectional enhanced-depth imaging spectral-domain optical coherence tomography (SDOCT) image in neurofibromatosis type 1. On the NIR image, white/grey rounded or patchy choroidal alterations typical of NF1 are evident and arrows indicate faint hyperreflective choroidal vessels. On the SDOCT cross-sectional image, arrows indicate dilated choroidal vessels.

NF1 is the most common disease in the phakomatoses, with a prevalence of 1 : 4000 individuals. This autosomal dominant disorder is diagnosed based on clinical findings established in 1988 by the National Institute of Health (NIH) consensus development statement. A minimum of 2 of the following major criteria are required for diagnosis: 6 or more café au lait spots, axillary or inguinal freckling, 2 or more cutaneous neurofibromas, 1 plexiform neurofibroma, distinctive osseous lesions, optic glioma, 2 or more iris Lisch nodules, and a first-degree relative with NF1 [11]. From the early 2000s, a few reports underlined the detection of choroidal abnormalities in this disorder using imaging methods [12, 13] and the first large cohort study was conducted in 2012 by Viola et al. [14]. Through the concomitant use of NIR imaging and SDOCT, hyperreflective rounded or patchy nodules were associated with hyperreflective signals in choroidal tissue, respectively (Figures 1 and 2). The prevalence of choroidal nodules detected by NIR was 71% in the paediatric population, a much higher frequency than the NIH ophthalmic diagnostic criteria of 43% for iris Lisch nodules [14]. Subsequent literature published on the topic confirmed NIR as a valid and reliable diagnostic imaging method in revealing choroidal abnormalities with a very high interobserver agreement in NF1 patients [15–17].

In recent years, Abdolrahimzadeh et al. described retinal microvasculature abnormalities (RVA) in NF1 using NIR imaging (Figure 1) [18]. These lesions were later confirmed in further studies on larger patient populations [16, 19]. RVA in NF1 are characterized by corkscrew and moyamoya-like vessel configurations with a tendency to increase with age. RVA have high diagnostic specificity with a positive predictive value of 100% [16]. A recently reported additional feature detected with NIR in NF1 patients is prominent choroidal vessels (Figure 2) [20].

NIR imaging is a sensitive, noninvasive, and reproducible examination that enables the detection of choroidal alterations, RVA, and enlarged choroidal vessels in NF1 patients. Choroidal alterations, detected with NIR, have been proposed as an additional diagnostic criterion in NF1 along with the original NIH criteria.

### 3.2. Tuberous Sclerosis Complex

Tuberous sclerosis complex (TSC), classified in the phakomatoses, is a multisystemic disease characterized by hamartomas that involve the central nervous system, eye, skin, heart, kidneys, liver, and lung. The estimated incidence and prevalence are 1/6800 and 1/15000, respectively, with 50% to 84% of sporadic cases [21]. Retinal astrocytic hamartoma (RAH) may be the first clinical sign and is a hallmark of TSC reported in 44% to 48% in two large case series [22, 23]. Diagnosis of typical RAH has traditionally been through fundus examination, but recent advances in imaging facilitate the detection of lesions using NIR and SDOCT [24].

**Figure 3:**
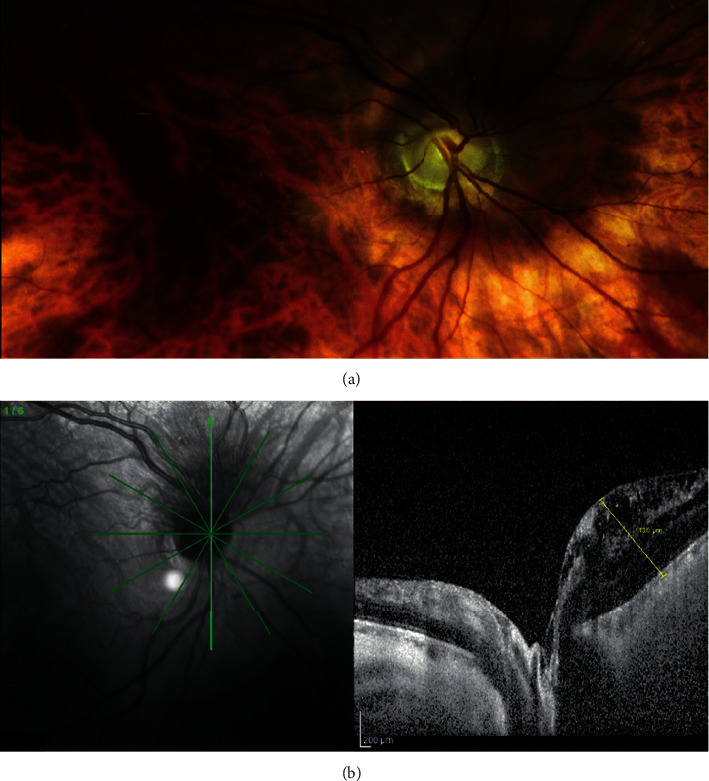
(a) Fundus image of the right eye. The fundus appearance is unremarkable, and retinal astrocytic hamartoma is not evident. (b) Near-infrared reflectance (NIR) and cross-sectional spectral-domain optical coherence tomography (SDOCT) image features of a peripapillary retinal astrocytic hamartoma (RAH) in the right eye of a patient with neurofibromatosis type 1. (a) NIR shows a shadowed area in the superior peripapillary margin of the optic disc; (b) SDOCT image at presentation shows the origin and expansion of a RAH in the retinal nerve fiber layer with numerous pinpoint and larger optically empty spaces within the mass; there is prominent outward bowing. The retinal nerve fiber layer, ganglion cell layer, and inner plexiform layer have considerable disorganization. The inner nuclear layer seems thickened. An initial splitting located in the outer plexiform layer is visible. The outer nuclear layer, external limiting membrane, and retinal epithelium are present. No calcification is present in the mass as there is no shadowing. The height of the mass is 1136 microns (modified with permission from Abdolrahimzadeh et al. [27]).

On ophthalmoscopic examination, RAH are divided into type 1 lesions that are flat and translucent or type 2 lesions that are elevated and multilobar with a “mulberry-like” appearance commonly associated with calcifications [23, 25]. Nyboer et al. reported 116 patients with TSC and described that translucent lesions could be easily overlooked on ophthalmoscopic examination due to only a slight difference in the background fundus color compared to lesions [23]. This difficulty in the detection of RAH can be overcome with NIR imaging, which reveals areas of hyperreflectivity that correspond to subtle thickening of the retinal nerve fiber layer (RNFL) or elevated honeycomb-like multicavitary intraretinal masses within the RNFL on SDOCT (Figure 3) [26–28].

Therefore, NIR and SDOCT are sensitive imaging modalities that enable detecting and localizing even small translucent RAH that are not readily visible on fundus ophthalmoscopy. This facilitates the diagnosis and follow-up of TSC patients where the examination is hindered by uncooperative patients or concomitant neurological or psychological conditions that characterize the disease.

### 3.3. Sturge–Weber Syndrome

**Figure 4:**
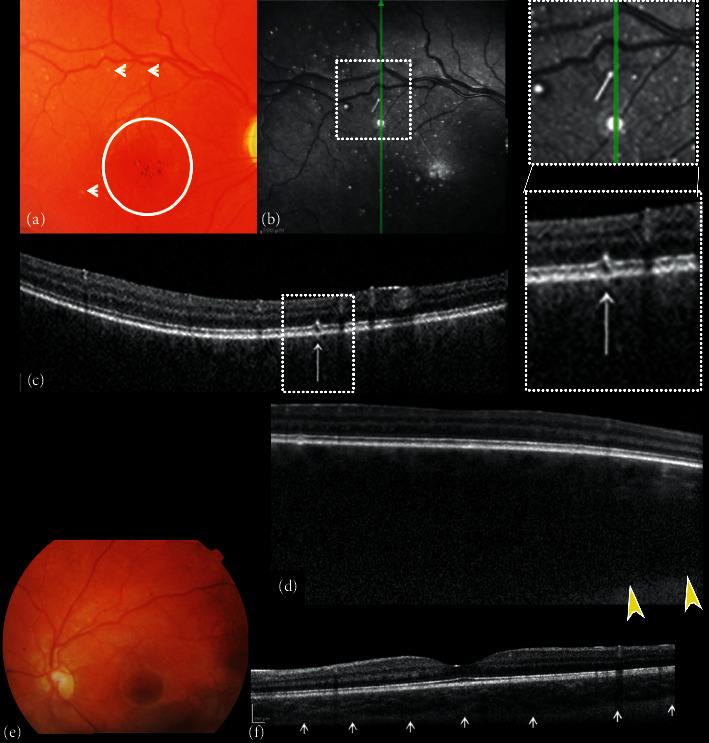
Composite of fundus color photographs and spectral-domain optical coherence (SDOCT) of the right and left eye. (a) The right fundus image shows excavation and pallor of the optic disk, absence of tessellation, diffuse choroidal hemangioma, hypo-hyper pigmentation of the foveal area with absent foveal reflex (circle), and small white dot shaped “microdrusen-like” alterations (arrows). (b) Near-infrared reflectance (NIR) of the right eye shows multiple hyperreflective dots surrounded by a hypo-reflective ring corresponding to the small white dot shaped “microdrusen-like” alterations of the posterior pole observed with ophthalmoscopy corresponded. B-scan cross-sectional SDOCT scan (c) on the hyperreflective dots shows focal alterations of the retinal pigment epithelial (RPE)-photoreceptor layer (involving the RPE, interdigital, ellipsoid and myoid zone), better seen on magnifications. (d) Enhanced-depth image (EDI) of the right eye shows choroidal thickness above 1000 *μ*m (yellow arrowheads indicate chorioscleral junction). (e) Left fundus image shows slightly increased excavation of the optic disc and absence of fundus tessellation. (f) EDI of the left eye shows no remarkable alterations; subfoveal choroidal thickness is 301 *μ*m (modified with permission from Abdolrahimzadeh et al [29]).

The Sturge–Weber syndrome (SWS) is a neuro-oculocutaneous disease characterized by leptomeningeal angiomatosis, ipsilateral facial naevus flammeus, congenital glaucoma, and diffuse choroidal hemangioma. SWS is diagnosed during infancy or childhood, where ophthalmological examination of patients is challenging and a fast and efficient imaging technique is fundamental. Enhanced-depth imaging SDOCT penetrates the RPE and enables assessing choroidal hemangiomas and correlated retinal complications. A recent report of an 8-year-old patient revealed a diffuse choroidal hemangioma characterized by multiple hyperreflective dots surrounded by hyporeflective rings on NIR. These alterations corresponded to focal alterations of the RPE-photoreceptor layer on SDOCT images and the white dot-shaped “microdrusen-like” alterations of the retina (Figure 4) [29].

### 3.4. Congenital Hamartomas

The congenital simple hamartoma of the RPE (CSHRPE) is a rare benign pigmented lesion found in phakomatoses [30]. The existing literature shows the role of NIR in delineating a hyperreflective lesion otherwise not clearly detectable on color fundus photography [31, 32]. Gass described this alteration as a hamartomatous malformation involving the RPE, retina, retinal vasculature, and overlying vitreous [33]. SDOCT enables appreciating the structure of these tumours in great detail. Furthermore, as the boundaries of combined hamartomas can be hard to distinguish clinically, NIR imaging facilitates delineating both the edges of the tumour and the extent of macular involvement in view of possible surgical management [34].

## 4. Hereditary Fundus Dystrophies

Hereditary fundus dystrophies commonly manifest during childhood and young adulthood resulting in profound visual impairment. As these diseases affect the RPE, photoreceptors, and the choriocapillaris complex, multimodal imaging is essential in the diagnostic work-up. NIR imaging is particularly indicated in patients with Bietti crystalline dystrophy (BCD) [8] and occult macular dystrophy, whereas NIR-autofluorescence (NIR-AF), with 787 nm excitation and 830 nm emission wavelength, is preferred in Best vitelliform and Stargardt dystrophy [35, 36].

### 4.1. Bietti Crystalline Dystrophy

Bietti crystalline dystrophy (BCD) is a rare autosomal recessive disease characterized by the deposition of crystalline material in the cornea, retina, RPE, and the choroid. CYP4V2 was identified as the causative gene in 2004 [37]. Retinal crystalline deposits are a characteristic feature in BCD; however, it is not always easy to differentiate these from other small deposits associated with other diseases. Diagnosis is particularly challenging when chorioretinal atrophy has progressed and only a few deposits are observed. Oishi et al. [8] considered NIR as the most practical imaging method in differentiating patients with BCD and CYP4V2 mutations from other chorioretinal dystrophies with crystalline-like retinal deposits. Although genetic testing is necessary to confirm the diagnosis, NIR is an essential first-step methodology with a sensitivity and specificity of 100% in identifying CYP4V2 mutation-positive patients. Histopathological studies show that the deposits in BCD originate from lipid crystallization [38] and hyperreflective crystalline formations are considered the SDOCT hallmark of cholesterol crystals as they present intense reflectivity with strong light scattering [5].

### 4.2. Occult Macular Dystrophy

Occult macular dystrophy (OMD) is hereditary dystrophy described by Miyake et al. in 1989 in patients with a bilateral progressive visual decline in the context of a normal fundus, fluorescein angiograms, and full-field electroretinogram (ERG) [39]. Retinitis pigmentosa 1-like 1 gene (RP1L1, OMIM 608581) is the only confirmed associated gene and dominant mutations in RP1L1 are responsible for occult macular dystrophy [40]. Focal macular and multifocal ERG are severely attenuated, indicating localized macular dysfunction [39]. NIR imaging reveals macular hyporeflective lesions easily discernible from the surrounding reflectance that correspond to an abnormal interdigitation and ellipsoid zone on SDOCT cross-sectional images. The hyporeflective alterations on NIR are confirmed in 66.7% of cases and are fundamental in diagnosis and monitoring disease progression [41].

### 4.3. Recessive Stargardt Disease

**Figure 5:**
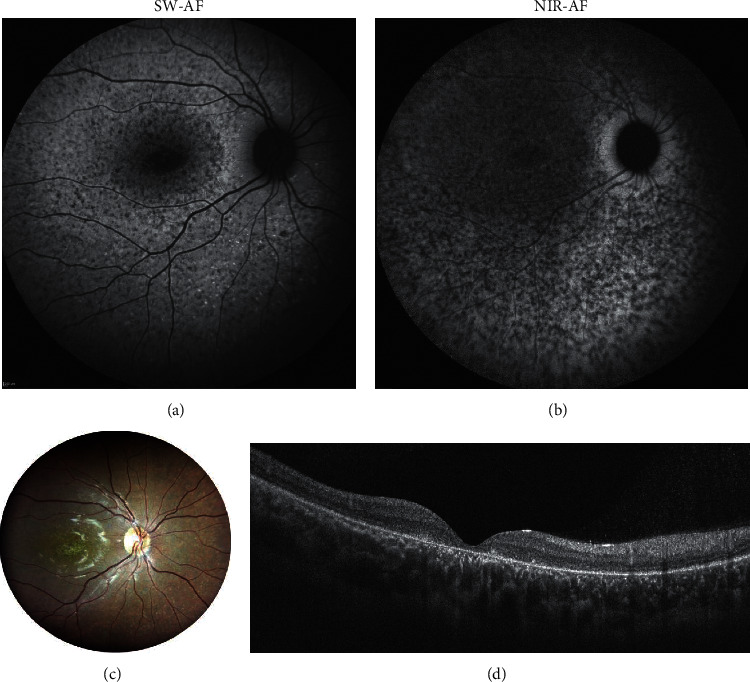
Composite images in a 19- year-old female with Stargardt disease. (a) Short-wavelength autofluorescence (SW-AF); (b) near-infrared autofluorescence (NIR-AF); (c) fundus photograph; (d) spectral-domain optical coherence tomography subfoveal b scan. Diffuse retinal pigment epithelium (RPE) atrophy is visible on both SW-AF and NIR-AF. RPE atrophy is more evident with NIR-AF exceeding the areas visible on SW-AF.

Recessive Stargardt disease occurs due to a deficiency of ABCA4 activity that accelerates the formation and accumulation of toxic bisretinoid molecules in RPE and photoreceptor cells. Multimodal imaging is central to this pathology and NIR-autofluorescence (NIR-AF) images, acquired using the indocyanine-green angiography mode (787 nm excitation), are particularly helpful in visualizing the RPE, which is commonly the first site of damage [35].  Figure 5 shows diffuse RPE atrophy visible on both short-wavelength (SW-AF) and near-infrared autofluorescence (NIR-AF) where lesions are more evident with NIR-AF in a 19-year-old female diagnosed with recessive Stargardt disease.

## 5. Other Macular Disorders

### 5.1. Acute Macular Neuropathy and Paracentral Acute Middle Maculopathy

Acute macular neuropathy (AMN) and paracentral acute middle maculopathy (PAMM) share similar clinical and multimodal imaging characteristics and NIR imaging represents the principle modality for diagnosis [42–44].

AMN is a rare acute pathology of uncertain aetiology presenting with a paracentral scotoma and characteristic wedge-shaped macular lesions, probably linked to oral contraceptive use in young women, prodromal viral infections, and shock [45]. On fundus photography, lesions appear as multiple dark brown areas pointing toward the fovea; however, these are often very faded and difficult to recognize. NIR clearly identifies hyporeflective, well-defined lesions that facilitate accurately monitoring lesion progression over time [46]. These hyporeflective lesions appear to correlate with SDOCT-hyperreflective alterations involving the outer plexiform/outer nuclear layer junction that precede outer segment abnormalities. Fawzi et al. hypothesized that optimal visualization of these lesions with NIR depends on persistent RPE melanin abnormalities [47].

The pathogenesis of AMN is still unclear; however, a microvascular ischemic insult is thought to be the precipitating risk factor [47] although the localization of the ischemic injury is still a matter of debate. In a study conducted by Thanos et al. [48], comparing NIR, SDOCT, and OCT angiography (OCTA), flow deficits were reported to be distributed at choriocapillaris level colocalizing with the hyporeflective lesions on NIR. Other authors hypothesized the deep capillary plexus as the prominent microvascular plexus affected in this condition [43, 49].

PAMM lesions can be associated with retinal vascular diseases. In young patients, a causative event may be represented by sickle cell crisis presenting with bilateral vision loss [42]. Color fundus photography shows nasal parafoveal whitening, NIR imaging reveals nasal parafoveal hyporeflective lesions, and SDOCT demonstrates hyperreflective changes affecting the middle retinal layers above the outer plexiform layer corresponding to the hyporeflective lesions on NIR [42, 50]. Similar to AMN, PAMM lesions correspond to hyperreflective bands localized in the inner plexiform and inner nuclear layers, accompanied by reduced flow in the intermediate and deep capillary plexuses [43, 50, 51].

### 5.2. Acute Idiopathic Maculopathy

In a case of acute idiopathic maculopathy presenting a bacillary layer detachment, NIR showed a hyperreflective perifoveal ring in the acute phase corresponding to subretinal fluid. Some residual central hyperreflectivity surrounded by a hyporeflective ring persisted following bacillary detachment resolution. This aspect has been related to a central thickening of the RPE/Bruch's membrane with adjacent ellipsoid and interdigitation zone disruption. In the case reported, NIR abnormalities persisted for months, even after fundus AF normalized [52].

### 5.3. Iatrogenic Laser-Induced Maculopathy

Retinal injuries secondary to laser pointers are increasingly frequent and different reviews have underlined how this has become a worrisome phenomenon. In 2017, Birtel et al. reported 111 cases of retinal injury due to laser pointers [53]. In 2018, Linton et al. documented 84 cases of handheld laser burns, where young boys represented the category most at risk [54]. The commonly reported symptoms following laser accidents include blurry vision, central scotomas, and reduced visual acuity. At fundoscopy, a multitude of retinal abnormalities can be observed spacing from circumscribed yellowish lesions, haemorrhage, pigment changes, and macular holes [53, 55]. However, lesions can be very subtle and easily missed, especially if the patient has difficulty in cooperating during the clinical examination or is reluctant to disclose the event and correlated symptoms. As laser-induced lesions primarily affect the photoreceptors in the interdigitation and ellipsoid zones with various degrees of RPE involvement, NIR is particularly useful in detecting these lesions [56].

**Figure 6:**
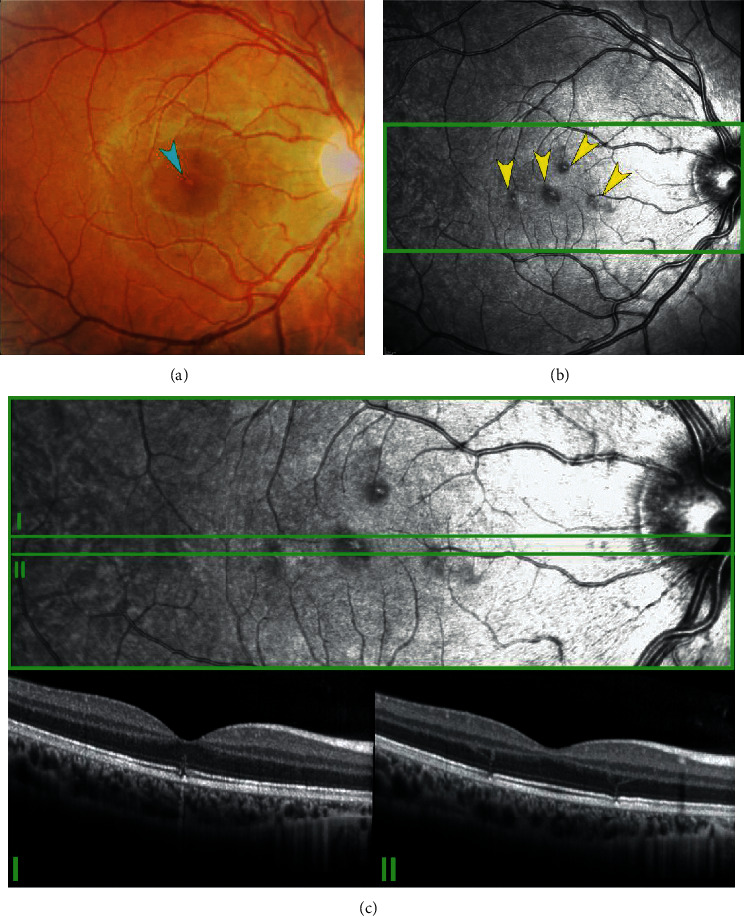
Multimodal imaging in a young patient with handheld laser-induced maculopathy. (a) Color fundus photograph shows a subtle focal yellowish-orange lesion in the foveolar area (light blue arrowhead). (b) Near-infrared reflectance demonstrates multiple roundish lesions with perilesional hyporeflective borders (yellow arrowheads). (c) SDOCT cross-sectional image scans through the lesions. (I-II) show defects of the ellipsoid zone and external limiting membrane with curvilinear hyperreflective tracts that appear to follow Henle fibers ascending toward the outer plexiform layer.

Herein, we report an illustrative case of a 16-year-old boy with a history of handheld laser misuse in his right eye for 15–20 seconds at 10 centimeters of distance. His best-corrected visual acuity was 20/20, and he denied visual disturbances. Clinical examination showed subtle macular changes. However, NIR enabled the identification of various parafoveal lesions with increased central reflectivity and hyporeflective borders that, on SDOCT cross-sectional images, corresponded to defects of the ellipsoid zone and external limiting membrane with curvilinear hyperreflective tracts (Figure 6).

A multimodal imaging approach including both NIR and SDOCT imaging in patients with suspected laser injury is imperative for diagnosis. Patients and parents benefit from counselling on the risks related to laser misuse in order to avoid exposure and repeated, possibly sight-threatening, damage.

## 6. Miscellaneous

Within the articles of this literature research a case of acute zonal cone photoreceptor outer segment loss in an adolescent boy was reported. Fundus examination, short-wavelength, and near-infrared FAF imaging were normal in both eyes, whereas NIR imaging showed a region of hyporeflectance that corresponded to a dense cone scotoma [57].

In early-onset cobalamin C (cblC) disease, patients show an early-onset, fast-progressing maculopathy with severe central outer nuclear and ganglion cell layer loss. In the context of multimodal imaging techniques, NIR showed bulls-eye maculopathy in a 14-month boy and was crucial for the interpretation of the SDOCT images that were acquired simultaneously [58].

Recent studies on dengue fever ocular manifestations demonstrated that the RPE is a target for the dengue virus and alterations in intercellular junctions following infection of RPE cells promote leakage of extracellular fluid into the retina. These lesions appear as crescent-shaped defects and are helpful in detecting subtle alterations on fundus examination [59, 60].

**Figure 7:**
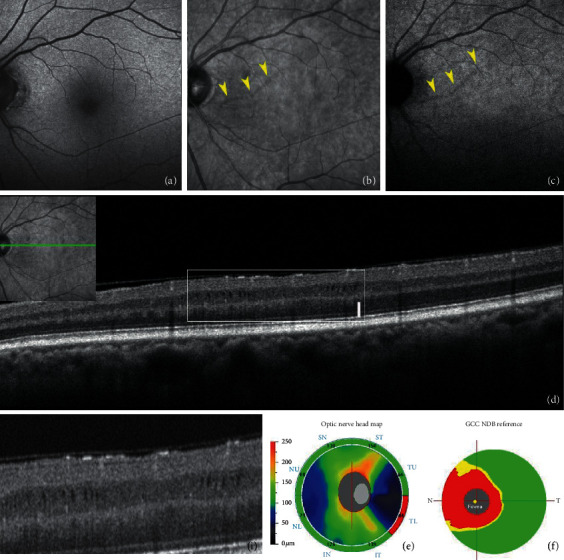
Microcystic edema. (a) Short-wavelength fundus autofluorescence (FAF) does not show alterations. (b) Infrared reflectance demonstrates a linear lesion of reduced reflectivity (yellow arrowheads), visible also at near-infrared FAF (yellow arrowheads (c)). (d) Spectral-domain optical coherence tomography b scan through the lesion as detailed in the miniature, the inset I reports a magnification of the microcystic spaces occupying the inner nuclear layer. (e) Retinal nerve fiber layer map confirming a defect in the inferior temporal sector and a diffuse defect in the ganglion cell layer map (f).

Microcystic macular edema occurring with optic neuropathies can be studied with multiple imaging techniques. NIR highlights well-circumscribed perifoveal retinal atrophic arcuate dark zones, which proved to have a very good correspondence both with cystic lesions in the inner nuclear layer on SDOCT scans and to scotomas detected with visual field examination (Figure 7) [61].

## 7. Conclusions

Near-infrared reflectance represents an optimal noninvasive imaging technique to visualize and enhance subretinal and sub-RPE alterations. This method can be combined with SDOCT cross-sectional scans to further improve diagnostic evaluation. The fast and straightforward acquisition mode is particularly useful in children who do not collaborate easily during ophthalmological examination. The role of NIR is essential in diagnosing several retinal diseases with onset from infancy to young adulthood. Beyond the difficulties in examining young patients, most of the alterations localized in the subretinal and sub-RPE space may be overlooked during a routine clinical examination. NIR is crucial in phakomatoses and facilitates the detection of choroidal alterations that are cardinal biomarkers in NF1. Another diagnostic application is the accurate visualization of the glistening crystals characterizing Bietti crystalline dystrophy. In AMN and PAMM lesions with young adulthood onset, NIR is not only diagnostic but also essential to monitor disease progression. An interesting clinical application is also the prompt recognition of laser-induced maculopathy where funduscopic examination can appear normal or subnormal.

In conclusion, NIR has a noninterchangeable role in the diagnosis of certain retinal and choroidal alterations, especially in children and young adults where a clinical examination is hindered owing to poor collaboration and lack of evident funduscopic findings. Moreover, this imaging method can reveal unique retinal biomarkers highly specific to rare conditions, which are otherwise difficult to recognize.

## Data Availability

The data supporting this narrative review were taken from previously reported studies and datasets, which have been cited. The processed data are available from the corresponding author upon request, e-mail: serena.fragiotta@uniroma1.it.
